# Towards best practice in acute stroke care in Ghana: a survey of hospital services

**DOI:** 10.1186/s12913-017-2061-2

**Published:** 2017-02-02

**Authors:** Leonard Baatiema, Michael Otim, George Mnatzaganian, Ama De-Graft Aikins, Judith Coombes, Shawn Somerset

**Affiliations:** 1 Regional Institute for Population Studies, University of Ghana, P.O Box LG96, Legon, Accra, Ghana; 2 School of Allied Health, Faculty of Health Sciences, Australian Catholic University, Sydney, Australia; 3 College of Health Sciences, University of Sharjah, Sharjah, United Arab Emirates; 4 College of Science, Health and Engineering, La Trobe Rural Health School, La Trobe University, Victoria, Australia; 5 School of Pharmacy, University of Queensland, Brisbane, Australia; 6 School of Allied Health, Faculty of Health Sciences, Australian Catholic University, Brisbane, Australia

**Keywords:** Stroke, Hospital services, Organised care, Evidence-based care, Health policy, Ghana

## Abstract

**Background:**

Stroke and other non-communicable diseases are important emerging public health concerns in sub-Saharan Africa where stroke-related mortality and morbidity are higher compared to other parts of the world. Despite the availability of evidence-based acute stroke interventions globally, uptake in low-middle income countries (LMIC) such as Ghana is uncertain. This study aimed to identify and evaluate available acute stroke services in Ghana and the extent to which these services align with global best practice.

**Methods:**

A multi-site, hospital-based survey was conducted in 11 major referral hospitals (regional and tertiary - teaching hospitals) in Ghana from November 2015 to April 2016. Respondents included neurologists, physician specialists and medical officers (general physicians). A pre-tested, structured questionnaire was used to gather data on available hospital-based acute stroke services in the study sites, using The World Stroke Organisation Global Stroke Services Guideline as a reference for global standards.

**Results:**

Availability of evidence-based services for acute stroke care in the study hospitals were varied and limited. The results showed one tertiary-teaching hospital had a stroke unit. However, thrombolytic therapy (thrombolysis) using recombinant tissue plasminogen activator for acute ischemic stroke care was not available in any of the study hospitals. Aspirin therapy was administered in all the 11 study hospitals. Although eight study sites reported having a brain computed tomographic (CT) scan, only 7 (63.6%) were functional at the time of the study. Magnetic resonance imaging (MRI scan) services were also limited to only 4 (36.4%) hospitals (only functional in three). Acute stroke care by specialists, especially neurologists, was found in 36.4% (4) of the study hospitals whilst none of the study hospitals had an occupational or a speech pathologist to support in the provision of acute stroke care.

**Conclusion:**

This study confirms previous reports of limited and variable provision of evidence based stroke services and the low priority for stroke care in resource poor settings. Health policy initiatives to enhance uptake of evidence-based acute stroke services is required to reduce stroke-related mortality and morbidity in countries such as Ghana.

**Electronic supplementary material:**

The online version of this article (doi:10.1186/s12913-017-2061-2) contains supplementary material, which is available to authorized users.

## Background

Stroke remains the second leading cause of deaths globally, recording a 26% increase in stroke deaths between 1990 and 2010 [1]. According to the World Stroke Society campaign highlights, one in six people in the world will suffer a stroke in their life time [2]. In Africa and other LMIC, stroke and other non-communicable diseases have become a great public health concern as current evidence suggests such settings are disproportionately affected by the overall global burden of stroke [3–5]. For example, of the estimated 5.9 million deaths linked to stroke worldwide in 2010, 71% were from LMIC settings [1]. Researchers further suggested that whereas high-income countries (HIC) show significant reductions in stroke incidence of about 42% over the last 40 years, a 100% increase in stroke incidence occurred in LMIC over the same period [6].

In Ghana, several studies have also confirmed an increasing stroke burden, with one month in-hospital case fatality as high as 41–43% [7–10]. For example, one of such studies examined a total of 12,233 stroke admissions over a 30 years period (1983–2013) and found that 28 day mortality during the study period was 41.1% [8]. In addition, the US Centres for Disease Control and Prevention (CDC) has reported that stroke is the fourth cause of mortality in Ghana [11]. These relatively high rates of in-hospital mortality raise important questions about the nature of acute stroke services in Ghana. Based on the current epidemiological transition attributed to aging populations, urbanization and modifiable stroke risk factors [12, 13] the future burden of stroke is substantial in LMIC [14], and inevitably, there will be an increased demand for acute stroke care interventions. The World Health Organisation (WHO) has previously asserted that the current stroke mortality burden in LMIC such as Ghana can be attenuated by the provision of quality and standardised stroke care [15], highlighting the need to ensure increased uptake to evidence-based stroke care interventions in such settings.

Despite the concept of evidence-based medicine is highly contested and has been diversely conceptualised [16, 17], the definition by Sackett et al. is widely acclaimed and accepted among medical researchers and practitioners [18]. The authors defined evidence-based medicine as ‘the conscientious, explicit, and judicious use of current best evidence in making decisions about the care of individual patients as well as integrating individual clinical expertise with the best available external clinical evidence from systematic research’. Thus, evidence-based acute stroke care interventions in this context refer to those interventions which are guided by sound scientific evidence, are well consistent with the clinical judgement and expertise of the individual clinician and meet the needs of patients for improved clinical outcomes.

Internationally, there is consistent body of evidence that stroke patients treated a) in a stroke unit by a multidisciplinary care team [19], b) using thrombolytic therapy through tissue plasminogen activator (t-PA) for acute ischemic stroke care patients within 4.5 h of stroke onset [20, 21], c) administering of aspirin for ischemic acute ischemic stroke patients within 48 h of a stroke [22], and d) decompressive surgery within 48 h of an acute stroke [23, 24] have reduced stroke-related mortality and morbidity. To support health systems worldwide especially of poor resource settings with high stroke burden to be able to consistently provide standard care for improved patient outcomes, a stroke services guideline by the World Stroke Organisation was developed [25].

Despite clear demonstration of the effectiveness of these evidence-based interventions for acute stroke care, there is widespread variation and limited uptake globally and this is substantially lower in LMIC such as Ghana [26–28]. The World Stroke Society has consequently prioritised access to evidence-based stroke care as a key theme in their 2016 global campaign, emphasizing the importance of this study. The slow uptake of evidence-based stroke care interventions is underpinned by multifactorial barriers at the health system, patient, health care providers and nature of acute stroke interventions levels [29–31].

To date, research on the availability of evidence-based acute stroke care interventions in hospital settings globally has been limited to high income countries. The UK, Australia, Canada and some European countries are exemplars [32–35]. Conversely, there is little information on evidence-based acute stroke services in Africa and other resource-poor settings. From the few studies available, the use of evidence-based acute stroke care services in LMIC settings is often asserted to be limited, poor and less likely to follow best practice guidelines [26–28, 36, 37]. An immediate question of public health interest will be to what extent are evidence-based acute stroke care interventions provided to acute stroke patients in the hospitals of LMIC such as Ghana? Addressing this question has the potential to provide baseline information on available evidence-based acute stroke care interventions and as well identify gaps in current stroke services which could support the development of future interventions seeking to standardise and improve acute stroke care services for optimal patient outcomes. This study therefore aims to identify available acute stroke services in Ghana and to evaluate the extent to which these services align with global best practice.

## Methods

### Study design and settings

This is descriptive study involving a survey conducted in major referral hospitals in all the ten administrative regions of Ghana to collect data on available acute stroke services from key acute stroke care providers between November 2015 and April 2016.

Ghana has a multi-health care system with the involvement of both public and private health care providers where healthcare delivery is provided by the formal medical healthcare system, faith-based health care system and the services from the ethno-medical system [38]. Of these, there are tertiary-teaching hospitals, regional hospitals, psychiatric hospitals, district hospitals and subdistrict health centres in the public sector [39]. However, the district hospitals and sub-district health centres often have limited clinical capacity for stroke care and so were excluded in this study. This study therefore purposively sampled only major public referral hospitals representing four of the five teaching hospitals as well as seven of the nine regional hospitals in Ghana. Except for one region (Greater Accra) where it was convenient to collect data from two major referral hospitals (one regional and one tertiary hospital), data was collected from either tertiary teaching or regional hospital which acted as the primary referral hospital in that particular administrative region. In spite of differences in the clinical capacities of the study hospitals on the basis of their status as a tertiary-teaching or regional hospital, these hospitals were chosen because they act as major referral hospitals for other hospitals and health centres in each of the ten administrative regions. Unlike the seven regional hospitals, the tertiary teaching hospitals serve as larger referral centres and are better-resourced with diagnostic and therapeutic facilities. They also serve as tertiary academic centres offering training in a range of highly specialised clinical disciplines. As presented in Table 1, the overall hospital bed capacity for admissions in the study sites ranged from 150 to 653 with annual stroke admissions for 2014 within the range of 49 to 1500 stroke cases per hospital.

**Table 1:** Characteristics of study hospitals and respondents

Hospital	2014 stroke admissions	Hospital bed capacity	Survey respondents
TH1	1500	653	Consultant Neurologist
TH2	1000	650	Neurologist
TH3	118	500	Physician Specialist
TH4	125	400	Medical Officer
RH1	409	194	Medical Officer
RH2	313	358	Medical Officer
RH3	-	250	Physician Specialist
RH4	520	235	Physician Specialist
RH5	71	400	Senior Medical Officer
RH6	39	226	Senior Medical Officer
RH7	49	200	Senior Medical Officer
Total			11

*TH* Tertiary (Teaching) Hospital, *RH* Regional Hospital

### Study participants

Respondents comprised neurologists, medical officers (general physicians) and physician specialists. These key informants were targeted because: they have relevant knowledge or expertise on acute stroke care in the study hospital; they play central roles as either acute stroke care providers or supervisors of the delivery of acute stroke care in the hospital. Their inclusion was also informed by the fact most play a role in making strategic decisions in the organisation of acute stroke care. All respondents were full time regular employees of the study sites. Part-time employees and resident physicians were not eligible. Administrative or other staff whose roles are not directly involved in the provision of acute stroke care to patients were also excluded.

### Sampling and recruitment

We employed a non-probabilistic purposive sampling technique to recruit respondents with one respondent per hospital. All study hospitals were formally contacted and their participation solicited using an official letter of invitation with information about the study, researchers and all ethical approval letters. The invitation letter also included a detailed study protocol and a study statement outlining the study purpose, potential study benefits, and an estimated time for survey completion. Prior to recruitment and actual data collection, discussions were held with the health administrators, clinical coordinators, regional human resource managers, medical directors and heads of department to select appropriate respondents. Consequently, one eligible respondent per study site was selected and contacted directly by the first author to organise the survey administration.

### Data collection

An interviewer-administered survey approach was conducted in respondents’ offices, clinic rooms and wards of each hospital. This approach was chosen to enhance response and compliance rates, provide opportunity for concepts and questions to be clarified and to ensure appropriate responses were elicited [40, 41].

The survey instrument (Additional file 1) was adapted from a previous national survey used in Australia [42]. A review of other relevant studies on acute stroke care quality indicators [25, 43–45] also informed the content of this instrument. The instrument was a paper-based, structured questionnaire containing 80-question items which required respondents to provide responses on the range of hospital-based services provided to acute stroke patients. The questionnaire was written and administered in English. Questions were mostly closed ended in nature and categorised into eight sections. These included information on respondents’ professional background and qualification, characteristics of study sites, institutional services and arrangements to support early presentation of acute stroke patients, and data on diagnosis, assessment services and stroke clinical guidelines. The remaining sections collected information on available acute stroke services and treatments, the stroke care workforce, key institutional policies, practices and interventions to support acute stroke care and on key challenges to acute stroke care.

### Quality control

The survey tool was pre-tested in six non-study sites among six acute stroke care physicians, representing two each from the southern, middle and northern belt of Ghana to account for the varied geo-political and socio-economic development differences. This process also tested the instrument’s suitability and relevance to the study settings, assess adequacy of response categories and whether all questions were appropriate within the study context [40, 41, 46]. The instrument was accordingly revised. Additionally, preliminary survey results were sent back to individual respondents for validation to ensure the data reflected their earlier responses.

### Data analysis

Analyses were conducted using the Statistical Package for the Social Sciences (SPSS) Version 22.0. Findings from the analysis were reported in the forms of numbers and percentages displayed in tables with scores underlying the availability of acute stroke care service and according to the study sites. Analysis also highlighted variances in available acute stroke services across the study hospitals. The World Stroke Society stroke service guideline [25] was used as a reference to evaluate the extent to which available services align with global best practice recommendations.

## Results

Respondents included four female and seven male acute stroke care providers, comprising Consultant Neurologist (1), Neurologist (1), Physician Specialist (3), Senior Medical Officer (3) and Medical Officer (3). Participants’ clinical practice experience ranged from three to twenty years. Table 1 presents the characteristics of the study hospitals and respondents. The next section presents the results from the survey as displayed in Table 2.

**Table 2:** Stroke services and availability in study hospitals

Thematic areas	Stroke services evaluated	Hospital response to available stroke services		
		Tertiary-Teaching Hospitals (*n* = 4)	Regional Hospital (*n* = 7)	Overall Total (*n* = 11) %
Acute Presentation of stroke	Accident and Emergency Department	4	7	11 (100.0%)
	Local emergency department protocols for rapid triage	1	0	1 (9.1%)
	Common means of stroke patient transport to hospital			
	− Local ambulance services	0	0	0 (0.0%)
	− Taxi/Private transport arrangement	4	7	11 (100.0%)
Diagnosis and Assessment Services	Functional CT Scan Service	3	4	7 (63.6%)
	CT scanner (24/7)	2	0	2 (18.2%)
	CT scanner (weekdays 9 am–5 am)	2	4	6 (54.5%)
	Functional MRI Scan Service	3	1	4 (36.4%)
	MRI (24/7)	0	0	0 (0.0%)
	MRI (weekdays 9 am–5 am)	3	1	4 (36.4%)
	Electrocardiogram (ECG)	4	5	9 (81.8%)
	Electroencephalogram	2	0	2 (18.2%)
	Neurovascular ultrasound diagnostic services e.g. Carotid Doppler Services	2	1	3 (27.3%)
	Magnetic Resonance Angiography	3	0	3 (27.3%)
	Computed Tomographic Angiography	4	4	8 (72.7%)
	National Institute of Health Stroke Scale (NIHSS)	2	2	4 (100.0%)
Acute Stroke services, treatments and rehabilitation services	Dedicated stroke unit (ward)	1	0	1 (9.1%)
	General (Medical) Ward	4	7	11 (100.0%)
	Multidisciplinary stroke care team	0	0	0 (0.0%)
	Thrombolytic therapy (t-PA)	0	0	0 (100.0%)
	Aspirin (antiplatelet)	4	7	11 (100.0%)
	Early discharge care plans	4	7	11 (100.0%)
	Revascularization (Carotid Endarterectomy)	0	0	0 (0.0%)
	Decompressive surgery (craniotomy)	0	0	0 (0.0%)
	Arteriovenous Malformation Treatment	0	0	0 (0.0%)
	Surgery for Aneurysm	0	0	0 (0.0%)
	On site rehabilitation services	4	7	11 (100.0%)
Stroke care workforce	Clinical psychologist	4	4	8 (72.7%)
	Trained Stroke Nurses	1	0	1 (9.1%)
	Physician Specialist	4	4	8 (72.7%)
	Neurosurgeon	3	0	3 (27.3%)
	Medical Officer	4	7	11 (100.0%)
	Nurse	4	7	11 (100.0%)
	Neurologist	3	1	4 (36.4%)
	Emergency department staff	4	7	11 (100.0%)
	Stroke care coordinator	0	0	0 (0.0%)
	Occupational therapist	0	0	0 (0.0%)
	Physiotherapist	4	7	11 (100.0%)
	Speech pathologist	0	0	0 (0.0%)
	Social worker	4	7	11 (100.0%)
	Dietician	4	7	11 (100.0%)
Health policy support for stroke care	Staff professional development and quality improvement for stroke care	0	0	0 (0.0%)
	National level support/ policies for stroke care			
	− High	0	0	0 (0.0%)
	− Average	0	0	0 (0.0%)
	− Low	0	0	0 (0.0%)
	− No support	4	7	11 (100.0%)
	Hospital level support/policies for stroke care			
	− High	0	0	0 (0.0%)
	− Average	0	2	2 (18.2%)
	− Low	2	1	3 (27.3%)
	− No support	2	4	6 (54.5%)
	Stroke register/Database	2	0	2 (18.2%)
	Community/hospital stroke awareness program	0	0	0 (0.0%)
	Access to community stroke rehabilitative programs	0	0	0 (0.0%)

Note: The listed numbers within the body of the tables indicate a “yes” answer

### Acute stroke presentation in hospitals

All participating hospitals have designated accident and emergency departments where acute stroke patients are first triaged. Only 9.1% of the hospitals reported the existence of locally developed protocols to support rapid triage of stroke patients. Both local ambulance services and private cars/taxis are used to transport acute stroke patients to hospital, with respondents indicating the predominant means of transport being commercial vehicles (taxi/cab). Local ambulance services were mostly used when patients were referred from another hospitals.

### Acute stroke diagnosis and assessment services

Available diagnostic and assessment services were diverse. Although 72.7% (8) of the study hospitals indicated the availability of a CT scan, only 63.6% (7) of these major referral hospitals had functional CT scan machines at the time of the survey. Access to brain CT scanning was only available 24 h/7 days in only 18.2% (2) of the study sites, whilst the rest only had access to these services during weekdays from 9 am–5 pm. Availability of MRI scan services and other advanced neurovascular diagnostic services such as electroencephalogram and interventional radiology were very limited (See Table 2). The survey showed only 36.4% (4) of the hospitals indicated the availability of MRI scanning services although only 27% (3) hospitals had functional MRI services. These services were only available only on weekdays from 9 am–5 pm. Carotid Doppler services were available in 3 hospitals with similar limited access during weekdays. None of the hospitals had specific stroke clinical guidelines. Instead, a general guideline for all health conditions known as the standard treatment guideline from which some guidelines on acute stroke care are embedded was available. Observations were made by the first author to ascertain the availability of CT and MRI brain scanning services in those study hospitals.

### Acute stroke care interventions and services

The survey revealed only 9.1% (1) of the study sites was reported to have a stroke unit. All hospitals had general medical wards for admissions and continuous in-patient care post-accident and emergency wards. Although elements of a multidisciplinary team for acute stroke care were evident, no functional and standardised one was reported in any of the hospitals. Furthermore, no provision of thrombolysis using tissue plasminogen activator for acute ischemic stroke care was reported in any of the study sites although 6 of respondents acknowledged awareness of this therapy. This absence was attributed to limited skilled personnel, cost and lack of national and organisational (hospital) level support to provide this therapy. In contrast, the use of aspirin for acute ischemic stroke was reported in all the hospitals. Surgical procedures for acute stroke care such as revascularization, decompressive craniotomy, arteriovenous malformation treatment, surgery for aneurysm treatment were not conducted in any of the study hospitals.

### Stroke care workforce

Specialist health workforce for acute stroke care was limited from the study. For example, the results showed only 36.4% (4) of the hospitals reported having a neurologist as part of the acute stroke care team whereas 27.3% (3) of the hospitals also indicated the availability of a neurosurgeon to their acute stroke care workforce. Other medical specialities (physician specialists) were reported in 72.7% (8) of the study sites. The availability of a speech therapists, occupational therapists and stroke nurses or stroke care coordinators was not reported in any of the study hospital. On the other hand, the availability of other acute stroke care staff in the study hospitals was within appreciable levels. All hospitals reported having medical doctors, nurses, physiotherapists and emergency department staff. In addition, clinical psychologists and social workers were also found in seven of the eleven hospitals.

### Health policy support for stroke care

Respondents indicated no direct health policy support from the state or national level for stroke care, or a national stroke policy framework, or national stroke clinical guideline existed. The common form of state support was a broad-based support for improvement of care across all hospital units and health conditions. Respondents reported that a national policy on non-communicable diseases existed but was yet to be operationalised as a full national policy framework due to lack of political will. On available opportunities for staff professional development and stroke care quality improvement programs, respondents reported that although all hospitals had policies to support staff develop professionally, these were not being implemented due to lack of funds. Only two hospitals had a stroke-specific database although all hospitals had a common database for all health conditions. No national level or hospital level community stroke awareness programs were reported.

## Discussion

To our knowledge, this is the first study to present information on the availability of best practice hospital level facilities for acute stroke services in Ghana. Importantly, the results from this study has clarified current uncertainties and speculation about the extent of evidence-based practice uptake for stroke care in a LMIC. Overall, the results suggest a limited adoption of evidence-based acute stroke care interventions, with the provision of a stroke unit care in only a single study hospital and no reported application of thrombolytic therapy as well as limited access to surgical procedures for acute stroke treatment. However, the use of aspirin for acute ischemic stroke was common to all study sites. Brain scanning services especially CT scans were often available but underutilised due to high cost of access. On the other hand, access to MRI and other advanced diagnostic services were more limited in the regional hospitals. Overall, national and hospital level health policy initiatives to support acute stroke care specifically were limited. This study also demonstrated that despite overall shortages in the specialist health workforce for acute stroke (especially neurologists), shortage of specialists were more predominant in the regional hospitals than the teaching hospitals.

Globally, emergency transport systems via ambulance and other emergency medical transport services are reported to support early and safe arrival for immediate provision of appropriate care to facilitate optimal patient health outcomes [47, 48]. However, the use of taxis or private cars was the commonest means of transport to hospitals by stroke patients in the present study, a situation which may lead to considerable delays in patient arrival for care. This has the potential to compromise prompt and safe responses for acute stroke patients in the major referral hospitals. Although this study reported the presence of a stroke unit in only one study hospital, this finding further corroborates previous reports indicating the limited provision of stroke unit care in LMIC [26, 27]. The stroke unit that was reported in this study is a six-bed capacity unit, found within a larger medical ward. Given the finding of only a single stroke unit of the eleven major referral hospitals in this study, it means the general medical wards represented the predominant acute stroke care wards despite evidence of less optimal patient outcomes compared to a stroke unit [19].

Additionally, evidence of no provision of thrombolytic therapy from the study finding supports earlier studies asserting the limited uptake of this treatment option in LMIC [28, 49, 50]. This requires policy attention to support the provision of thrombolytic therapy in these major referral hospitals in view of the substantial effects of this therapy on stroke survival. The widespread use of aspirin therapy by all study hospitals for acute stroke care may reflect this intervention as an inexpensive choice and easy to administer in clinical settings [51, 52]. The uptake of aspirin could further be maximised with enhanced access to brain scanning devices to ensure eligible patients are treated with aspirin.

Although seven of eleven study sites provided brain CT scan services, access was limited only to weekdays (9 am–5 pm). This has important implications for early access to appropriate care given that improved patient outcomes are often time-dependent [53]. This highlights the need for improved access to brain imaging services in hospitals, especially regional, because most stroke patients are likely to be treated in non-teaching hospitals before they are referred to tertiary teaching hospitals. The importance of this is heightened by the fact that patient referral to such tertiary hospitals is however largely dependent on the financial capacity of the patient. Although there is still lack of clarity on the direct influence of access to brain CT scanning services on mortality and morbidity, without early access to brain scan services, there is high potential for inappropriate provision of care to patients (e.g. inadvertent administration of aspirin therapy to a haemorrhagic stroke patient).

The considerable deficit in the human resource capacity to treat acute stroke patients in all study sites, especially in regional hospitals, is also noteworthy. Acute stroke care by a neurologist was limited to four of eleven study hospitals whilst care provided by occupational and speech therapists were not reported in any of the hospitals. This gap will likely inhibit effective patient evaluation for impairment and disability. For example, given that the incidence of dysphasia following an acute stroke is around 37–78% [54], the absence of speech therapist to conduct effective assessment and support likely compromises the quality of life of stroke patients with dysphasia. These results reaffirm the limited availability of acute stroke care workforce asserted in previous studies [55–57]. In a key report [57] outlining the principal challenges compromising global efforts to control the increasing burden of non-communicable diseases such as stroke, the issue of inadequate skilled health work force was highlighted. This indicates an important gap in the capacity of the health care systems to provide acute stroke care, although regional hospitals require more policy attention, particularly via rehabilitative specialists, neurologists, neurosurgeons and other stroke specialists to support optimal recovery in view of reduced in-patient stroke mortality and dependency at discharge following treatment by neurologists [58]. As demonstrated in Nigeria [59], in sub-Saharan Africa, a study on task shifting of specialist stroke work force roles to non-specialists demonstrated improved knowledge on acute stroke care and so this could be explored by the Ghanaian health policy makers and managers as a short term measure to address the current stroke workforce deficit.

The absence of national level support for acute stroke care, limited funding from hospital management, no community/hospital stroke awareness program, and state supported community stroke rehabilitative programs reported in this study raises important health policy questions about the state’s readiness and commitment to reducing the increasing stroke burden. Long-term and community-level care after in-patient care remains largely a family responsibility in the absence of a community level rehabilitative care. This augments earlier reports of limited health policy commitment to address the current disease burden posed by stroke and other non-communicable diseases in Ghana [39, 60–63]. It also concurs with reports of low prioritization of stroke care in the health policy agendas of most LMIC [64–67]. Contextualising this within the global health policy support for stroke, the deficit in health policy support in this study underscores previous reports of inadequate global health funding for stroke and other non-communicable diseases [3, 68]. For example, the Institute for Health Metrics and Evaluation recently reported that only 1.3% of overall donor support for health was allocated to NCDs in 2015 [68]. As demonstrated in some other LMIC [69, 70], the capacity of Ghanaian health systems could be improved to address the growing stroke burden by increasing funding and infrastructural support, training for more stroke specialists, formulating evidence-informed policies, plans, treatment guidelines and strategies to support effective acute stroke diagnosis and treatment.

The historical development gap between northern Ghana and the rest was also evident in the inequitable distribution of stroke services across the study sites. Overall, the tertiary hospitals were much more equipped with modern stroke services to support standard care compared to the regional hospitals. However, the major referral hospitals in the northern part of Ghana recorded limited evidence-based acute stroke services. For example, apart from the single tertiary hospital in northern Ghana, the other two northern regional hospitals do not have CT, MRI brain scanning services or a neurologist. This finding reinforces earlier reports of limited access to health care facilities in the northern parts of Ghana and has often resulted in poor health outcomes compared to the other parts of Ghana [39, 71, 72]. To address this, an affirmative action in the form of health policy reforms to address this situation will be in the right direction.

Based on the global stroke services guideline for stroke care proposed by the World Stroke Society [25] as reported in Table 3, this study suggests an overall limitation in the capacity of the health care system in both teaching and regional hospitals to provide advanced evidence-based acute stroke services. The present study results shows the major tertiary - teaching hospitals have more capacity to provide most of the essential elements of the evidence-based acute stroke care recommendation compared to the regional hospitals despite the fact that regional hospitals first receive most of the acute stroke cases before likely referring to a teaching hospital. In general, the current results underline the overarching scope and need to improve evidence-based practice for acute stroke care in resource poor countries such as Ghana.

**Table 3:** World Stroke Organization checklist for health service capacity for acute stroke care

Component of acute stroke service	Service availability		
	Tertiary-Teaching Hospitals (*n* = 4)	Regional Hospital (*n* = 7)	Overall Total (*n* = 11) %
Advanced stroke services			
Access to advanced diagnostic services			
− Magnetic Resonance Angiography	3	0	3 (27.3%)
− Computed Tomographic Angiography	4	4	8 (72.7%)
− Electroencephalogram	0	0	0 (0.0%)
− Electrocardiogram (ECG)	4	5	9 (81.8%)
− Neurovascular ultrasound diagnostic services, e.g. Carotid Doppler Services	3	0	3 (27.3%)
− Magnetic Resonance Imaging	4	1	5 (45.5%)
− Computed Tomographic Scan	4	4	8 (72.7%)
Access to physicians with stroke expertise (and physician specialists)			
− Neurologists	3	1	4 (36.4%)
− Neurosurgeon	3	0	3 (27.3%)
− Physician Specialist	4	4	8 (72.7%)
Access to advanced acute stroke care interventions			
− Stroke unit care	1	0	1 (9.1%)
− Tissue plasminogen activator (t-PA)	0	0	0 (0.0%)
− Decompressive surgery	0	0	0 (0.0%)
− Arteriovenous Malformation Treatment	0	0	0 (0.0%)
Surgery for Aneurysm	0	0	0 (0.0%)
Revascularization (Carotid Endarterectomy)	0	0	0 (0.0%)
Access to specialist rehabilitation therapists			
− Physiotherapists	4	7	11 (100.0%)
− Occupational Therapists	0	0	0 (0.0%)
− Speech Therapists	0	0	0 (0.0%)
Access to community programs for recovery after stroke	0	0	0 (0.0%)
Essential stroke services			
Access to basic diagnostic services			
− Laboratory	4	7	11 (100.0%)
− ECG	4	5	9 (81.8%)
− Computed Tomographic Scan (CT scan)	4	4	8 (72.7%)
− Neurovascular ultrasound diagnostic services	3	0	3 (27.3%)
− National Institutes of Health Stroke Scale (NIH)	2	2	2 (18.2%)
Access to nurses	4	7	11 (100.0%)
Access to physicians, not necessarily stroke specialists	4	7	11 (100.0%)
Access to acute thrombolysis with t-PA	0	0	0 (0.0%)
Access to stroke unit care	1	0	1 (9.1%)
Antiplatelet (Aspirin) therapy	4	7	11 (100.0%)
Access to rehabilitation services	4	7	11 (100.0%)
Minimal healthcare services			
Variable access to healthcare workers (nurses or lay workers)	4	7	11 (100.0%)
Very limited access to physicians	0	2	2 (18.2%)
No access to diagnostic services or hospital care	0	0	0 (0.0%)
Care provided in local communities	0	0	0 (0.0%)

Note: The listed numbers within the body of the tables indicate a “yes” answer

### Strengths and limitations

This study is the first to inform on availability of evidence-based acute stroke care interventions in Ghana. An added strength relates to its focus on regional and tertiary-teaching hospitals being the major referral hospitals in each administrative region in Ghana, gives the study a national character with potential generalizable results and relevance to other low-middle income settings. However, this was a descriptive study which limited our ability to evaluate the effects of these services on patient clinical outcomes. This study was also limited in scope by restriction to eleven public regional and teaching hospitals and excluding private and non-regional hospitals. Future studies should focus on both public and private hospitals to achieve better representation.

## Conclusion

A growing body of evidence on effective acute stroke care interventions exist. However, results from this study highlight limited access to these services especially in the regional non-tertiary teaching hospitals. Significant effort is required to ensure acute stroke patients access the best care not only in tertiary but also regional hospitals given that not all acute stroke patients can afford care in teaching hospitals.

Based on this study, it is clear Ghana is yet to adequately translate in health policy wise its global commitments to reducing the global burden of stroke. An overall improvement in national policy for stroke care is needed. This should be well targeted and equity-based given the significant disparities found across these major referral hospitals in our study. With current projections of a global rise in stroke incidence and the increasing aging population, demand for acute stroke care will inevitably witness further increase and so there is considerable scope to improve acute stroke care in low middle income countries such as Ghana in order to minimise premature stroke-related mortality and disability.

## Additional file

Additional file 1: Survey Instrument: Acute Stroke Care Services in Ghanaian Hospitals (DOCX 40 kb)

## Abbreviations

CDC: Centres for Disease Control and Prevention
CT: Computed Tomographic
HIC: High-Income Countries
LMIC: Low-Middle Income Countries
MRI: Magnetic Resonance Imaging
NCDs: Non-Communicable Diseases
SPSS: Statistical Package for the Social Sciences
t-PA: Tissue Plasminogen Activator
WHO: World Health Organisation

## References

[1] Mensah GA, Norrving B, Feigin VL (2015). The global burden of stroke. Neuroepidemiology.

[2] Kaste M, Norrving B (2010). Editorials: from the world stroke day to the world stroke campaign: one in six: act now!. Int J Stroke.

[3] Daniels ME, Donilon TE, Bollyky TJ (2014). The emerging global health crisis: noncommunicable diseases in low-and middle-income countries. Council on Foreign Relations Independent Task Force Report.

[4] Bloom DE, Cafiero E, Jané-Llopis E, Abrahams-Gessel S, Bloom LR, Fathima S (2012). The global economic burden of noncommunicable diseases.

[5] Owolabi MO, Akarolo-Anthony S, Akinyemi R, Arnett D, Gebregziabher M, Jenkins C (2015). The burden of stroke in Africa: a glance at the present and a glimpse into the future. Cardiovasc J Afr.

[6] Feigin VL, Lawes CM, Bennett DA, Barker-Collo SL, Parag V (2009). Worldwide stroke incidence and early case fatality reported in 56 population-based studies: a systematic review. Lancet Neurol..

[7] Agyemang C, Attah-Adjepong G, Owusu-Dabo E, De-Graft Aikins A, Addo J, Edusei AK (2012). Stroke in Ashanti region of Ghana. Ghana Med J.

[8] Sarfo FS, Akassi J, Awuah D, Adamu S, Nkyi C, Owolabi M (2015). Trends in stroke admission and mortality rates from 1983 to 2013 in central Ghana. J Neurol Sci.

[9] Sarfo FS, Awuah DO, Nkyi C, Akassi J, Opare-Sem OK, Ovbiagele B (2016). Recent patterns and predictors of neurological mortality among hospitalized patients in Central Ghana. J Neurol Sci.

[10] Sarfo F, Acheampong J, Oparebea E, Akpalu A, Bedu-Addo G (2014). The profile of risk factors and in-patient outcomes of stroke in Kumasi, Ghana. Ghana Med J.

[11] Centers for Disease Control and Prevention. Centers for Disease Control and Prevention, Ghana Factsheet. Atlanta; 2013. Report No. https://www.cdc.gov/globalhealth/countries/ghana/pdf/ghana-2013.pdf.

[12] Feigin VL, Forouzanfar MH, Krishnamurthi R, Mensah GA, Connor M, Bennett DA (2014). Global and regional burden of stroke during 1990–2010: findings from the Global Burden of Disease Study 2010. Lancet.

[13] Roth GA, Forouzanfar MH, Moran AE, Barber R, Nguyen G, Feigin VL (2015). Demographic and epidemiologic drivers of global cardiovascular mortality. N. Engl. J. Med..

[14] Strong K, Mathers C, Bonita R (2007). Preventing stroke: saving lives around the world. Lancet Neurol..

[15] Asplund K (2005). What MONICA told us about stroke. Lancet Neurol..

[16] Greenhalgh T, Howick J, Maskrey N. Evidence based medicine: a movement in crisis? BMJ. 2014;348:g3725. 10.1136/bmj.g3725 PMC4056639 24927763

[17] Dopson S, Fitzgerald L (2005). Knowledge to action?: evidence-based health care in context.

[18] Sackett DL, Rosenberg W, Gray J, Haynes RB, Richardson WS (1996). Evidence based medicine: what it is and what it isn’t. BMJ.

[19] Stroke Unit Trialists' Collaboration. Organised inpatient (stroke unit) care for stroke. Cochrane Database Syst Rev. 2013(9). Art. No.: CD000197. doi: 10.1002/14651858.CD000197.pub3. 10.1002/14651858.CD000197.pub3 PMC6474318 24026639

[20] Wardlaw JM, Murray V, Berge E, del Zoppo GJ. Thrombolysis for acute ischaemic stroke. The Cochrane Library; 2014. 10.1002/14651858.CD000213.pub3 PMC4153726 25072528

[21] Hacke W, Kaste M, Bluhmki E, Brozman M, Dávalos A, Guidetti D (2008). Thrombolysis with alteplase 3 to 4.5 hours after acute ischemic stroke. N. Engl. J. Med..

[22] Sandercock PAG, Counsell C, Tseng MC, Cecconi E. Oral antiplatelet therapy for acute ischaemic stroke. Cochrane Database Syst Rev. 2014;(3): Art. No.: CD000029. doi: 10.1002/14651858.CD000029.pub3. 10.1002/14651858.CD000029.pub3 PMC6669270 24668137

[23] Donnan GA, Davis SM, Parsons MW, Ma H, Dewey HM, Howells DW (2011). How to make better use of thrombolytic therapy in acute ischemic stroke. Nat Rev Neurol.

[24] Vahedi K, Hofmeijer J, Juettler E, Vicaut E, George B, Algra A (2007). Early decompressive surgery in malignant infarction of the middle cerebral artery: a pooled analysis of three randomised controlled trials. Lancet Neurol..

[25] Lindsay P, Furie KL, Davis SM, Donnan GA, Norrving B (2014). World Stroke Organization Global Stroke Services guidelines and action plan. Int J Stroke.

[26] Brainin M, Teuschl Y, Kalra L (2007). Acute treatment and long-term management of stroke in developing countries. Lancet Neurol..

[27] Langhorne P, de Villiers L, Pandian JD (2012). Applicability of stroke-unit care to low-income and middle-income countries. Lancet Neurol..

[28] Berkowitz AL, Mittal MK, McLane HC, Shen GC, Muralidharan R, Lyons JL (2014). Worldwide reported use of IV tissue plasminogen activator for acute ischemic stroke. Int J Stroke.

[29] Dale S, Levi C, Ward J, Grimshaw JM, Jammali-Blasi A, D’Este C (2015). Barriers and enablers to implementing clinical treatment protocols for fever, hyperglycaemia, and swallowing dysfunction in the quality in acute stroke care (QASC) project-a mixed methods study. Worldviews Evid Based Nurs.

[30] Meurer WJ, Majersik JJ, Frederiksen SM, Kade AM, Sandretto AM, Scott PA (2011). Provider perceptions of barriers to the emergency use of tPA for acute ischemic stroke: a qualitative study. BMC Emerg Med.

[31] Williams JM, Jude MR, Levi CR (2013). Recombinant tissue plasminogen activator (rt‐PA) utilisation by rural clinicians in acute ischaemic stroke: a survey of barriers and enablers. Aust. J. Rural Health.

[32] Leys D, Ringelstein EB, Kaste M, Hacke W (2007). Initiative ECotES. Facilities available in European hospitals treating stroke patients. Stroke.

[33] Network CS (2011). The quality of stroke care in Canada.

[34] National Stroke Foundation. National Stroke Audit – Acute Services Report. Melbourne: National Stroke Foundation; 2015.

[35] Paley L, Kavanagh S, Kavanagh M (2014). Sentinel Stroke National Audit Programme (SSNAP) Clinical Audit January-March 2014 Public Report.

[36] Kengne AP, Anderson CS (2006). The neglected burden of stroke in Sub‐Saharan Africa. Int J Stroke.

[37] Norrving B, Kissela B (2013). The global burden of stroke and need for a continuum of care. Neurology.

[38] Aikins Ad-G. Chronic Non-communicable Diseases in Ghana.: Multidisciplinary Perspectives. Accra: Sub-Saharan Publishers; 2014.

[39] Saleh K (2012). World Bank study: a health sector in transition to universal coverage in Ghana.

[40] Rea LM, Parker RA. Designing and conducting survey research: A comprehensive guide. San Francisco: Wiley; 2012.

[41] Kelley K, Clark B, Brown V, Sitzia J (2003). Good practice in the conduct and reporting of survey research. International J Qual Health Care.

[42] National Stroke Foundation (2013). National Stroke Audit - acute services organisational survey report.

[43] Purvis T, Cadilhac D, Donnan G, Bernhardt J (2009). Systematic review of process indicators: including early rehabilitation interventions used to measure quality of acute stroke care. Int J Stroke.

[44] Wolfe C, McKevitt C, Rudd A (2002). Stroke services: policy and practice across.

[45] Hall RE, Khan F, Bayley MT, Asllani E, Lindsay P, Hill MD (2013). Benchmarks for acute stroke care delivery. International J Qual Health Care.

[46] Bowling A (2014). Researhc methods in health: investigating health and health services.

[47] Mosley I, Nicol M, Donnan G, Patrick I, Kerr F, Dewey H (2007). The impact of ambulance practice on acute stroke care. Stroke.

[48] Harbison J, Massey A, Barnett L, Hodge D, Ford GA (1999). Rapid ambulance protocol for acute stroke. Lancet.

[49] Durai Pandian J, Padma V, Vijaya P, Sylaja P, Murthy JM (2007). Stroke and thrombolysis in developing countries. Int J Stroke.

[50] Ghandehari K. Barriers of Thrombolysis Therapy in Developing Countries. Stroke Res Treat. 2011;2011:686797. doi: 10.4061/2011/686797. 10.4061/2011/686797 PMC3095908 21603174

[51] Gaziano TA (2005). Cardiovascular disease in the developing world and its cost-effective management. Circulation.

[52] Gilligan AK, Thrift AG, Sturm JW, Dewey HM, Macdonell RAL, Donnan GA (2005). Stroke units, tissue plasminogen activator, aspirin and neuroprotection: which stroke intervention could provide the greatest community benefit?. Cerebrovasc Dis.

[53] Saver JL (2006). Time is brain—quantified. Stroke.

[54] Martino R, Foley N, Bhogal S, Diamant N, Speechley M, Teasell R (2005). Dysphagia after stroke incidence, diagnosis, and pulmonary complications. Stroke.

[55] El Khamlichi A (2001). African neurosurgery: current situation, priorities, and needs. Neurosurgery.

[56] Chin JH (2012). Stroke in sub-Saharan Africa: an urgent call for prevention. Neurology.

[57] Daar AS, Singer PA, Persad DL, Pramming SK, Matthews DR, Beaglehole R (2007). Grand challenges in chronic non-communicable diseases. Nature.

[58] Goldstein L, Matchar D, Hoff-Lindquist J, Samsa G, Horner R (2003). VA Stroke Study Neurologist care is associated with increased testing but improved outcomes. Neurology.

[59] Akinyemi RO, Owolabi MO, Adebayo PB, Akinyemi JO, Otubogun FM, Uvere E (2015). Task-shifting training improves stroke knowledge among Nigerian non-neurologist health workers. J Neurol Sci.

[60] World Health Organization. Noncommunicable Diseases Country Profile 2014. Geneva: WHO; 2014.

[61] Bosu W (2013). A comprehensive review of the policy and programmatic response to chronic non-communicable disease in Ghana. Ghana Med J.

[62] Aikins A-G, Boynton P, Atanga LL (2010). Review developing effective chronic disease interventions in Africa: insights from Ghana and Cameroon.

[63] Ama de-Graft Aikins, Samuel Agyei-Mensah, Charles Agyemang. Chronic Non-communicable Diseases in Ghana. Multidisciplinary Perspectives (Vol. 1). Sub-Saharan Publishers; 2013.

[64] Cadilhac D, Lalor E, Pearce D, Levi C, Donnan G (2006). Access to stroke care units in Australian public hospitals: facts and temporal progress. Intern Med J.

[65] Rudd A, Hoffman A, Down C, Pearson M, Lowe D (2007). Access to stroke care in England, Wales and Northern Ireland: the effect of age, gender and weekend admission. Age Ageing.

[66] World Health Organization (2005). Preventing chronic diseases: a vital investment: WHO global report.

[67] Fuster V, Voute J, Hunn M, Smith SC (2007). Low priority of cardiovascular and chronic diseases on the global health agenda a cause for concern. Circulation.

[68] Institute for Health Metrics and Evaluation (2016). Financing Global Health 2015: development assistance steady on the path to new Global Goals.

[69] Atun R, Jaffar S, Nishtar S, Knaul FM, Barreto ML, Nyirenda M (2013). Improving responsiveness of health systems to non-communicable diseases. Lancet.

[70] Byfield S, Moodie R (2013). Addressing the world’s biggest killers: Non-communicable diseases and the international development agenda.

[71] Atuguba RA (2013). The right to health in Ghana: healthcare, human rights, and politics.

[72] Ghana Health Service. 2014 Annual Report. Accra: GHS; 2015. Available from: https://www.ghanahealthservice.org/downloads/Ghana_Health_Service_2014_Annual_Report.pdf.

[73] General Assembly of the World Medical Association (2014). World Medical Association Declaration of Helsinki: ethical principles for medical research involving human subjects. J Am Coll Dent.

