# Supplementary material for: Towards best practice in acute stroke care in Ghana: a survey of hospital services
Source: BMC Health Serv Res. 2017 Feb 2;17:108. doi: 10.1186/s12913-017-2061-2 (PMC5290633; doi:10.1186/s12913-017-2061-2)
Supplement: Additional file 1: — Survey Instrument: Acute Stroke Care Services in Ghanaian Hospitals (DOCX 40 kb) [file 12913_2017_2061_MOESM1_ESM.docx]

**Survey Instrument: Acute Stroke Care Services in Ghanaian Hospitals**

|  | **SECTION A: RESPONDENT'S INFORMATION** | |
| --- | --- | --- |
| 1 | Position of Respondent | …………………………………………………………………………………………………………………… |
| 2 | Gender | Male...……………………………....………………..1  Female ……………………………………..………...2 |
| 3 | Years of work experience | 0-5yrs………………………………………….…..…1  6-10yrs………………………….…………….….…...2  11-15yrs………………………….………….……..…3  16years and above………………………………..…..4 |
|  | **SECTION B: HOSPITAL DATA** | |
| 4 | Name of Hospital | …………………………………………………………………………………………………………………… |
| 5 | Status of Hospital | Regional Hospital…………………………….…..….1  Teaching Hospital……………..……………….…… 2 |
| 6 | Number of beds in the hospital | ……………………………………………………………………….………………………………………….. |
| 7 | How many acute stroke patients were admitted in your hospital in the last year (2015) | ……………………………………………………………………………………………………………………………………………………………………………… |
|  | **SECTION C: ACUTE PRESENTATION AND EARLY ASSESSMENT** | |
| 8 | Which ward is a patient with acute stroke symptoms most likely to be admitted into first? | Accident and Emergency Department……………......1  General medical ward…………………………….......2  Neurology ward.……………………….…………......3  Intensive Care Unit (ICU)…………………...…….…4  Medical Ward…………………………………...……5  If others, specify...………….…………..………….....6 |
| 9 | Are there accident and emergency department protocols for rapid triage for patients presenting with acute stroke in your hospital? | Yes …………………..…………………………..…...1  No …………………………………………..….…….2 |
| 10 | Are there arrangements in place with local ambulance services to fast-track patients presenting with acute stroke? | Yes……………………………………………………1  No…………………………………………………….2 |
| 11 | If no to Q10, what is the common means of transport for rapid patient transfer to your hospital? | Taxi /private arrangements…………………………...1  Others………………….………………………...…...2  If others, please specify……………………………… |
| 12 | Are there protocols for transfer of stroke patients to other hospitals for care? | Yes……………………………………………………1  No…………………………………………………….2  Don’t know………………………………….…...…...3 |
| 13 | Are there clinical management guidelines for acute stroke care | Yes ………………………………..………………….1  No……..……………..…………………….…………2 |
| 14 | If yes to Q13, which clinical management guidelines is/are used for acute stroke care in the hospital? | National clinical guideline for stroke…………….…..1  Standard Treatment Guideline…………….………….2  World Stroke Society Clinical guideline…………......3  NICE Clinical Guideline……………………...……...4  If others, specify ……………………………………..5 |
|  | **SECTION D: DIAGNOSIS AND SCREENING SERVICES** | |
| 15 | Does your health service have access to functional CT scanner? | Yes ………………………………………………..…1  No …………..……………………....…………….…2 |
| 16 | If yes to Q15, what hours is the service available? (Select one option only) | Monday – Friday, 9am – 5pm…………………….….1  Monday – Friday, extended hours……………….…...2  Extended hours including weekends……..…………..3  24 hours a day, 7 days a week ……………….………4 |
| 17 | If no to Q15, can you get CT offsite within 24 hours of stroke presentation to your hospital? | Yes …………………………….………..………...….1  No ……………………………………….………...…2 |
| 18 | Does your health service have access to functional MRI services? | Yes ……………………………………….………….1  No …………………………………..…….…………2 |
| 19 | If yes to Q18,what hours is the service available? (Select one option only) | Monday – Friday, 9am – 5pm………………….…….1  Monday – Friday, extended hours…………….……..2  Extended hours including weekends………….……..3  24 hours a day, 7 days a week ………………………4 |
| 20 | If no to Q18, can you get MRI offsite within 24 hours of stroke presentation to your hospital? | Yes……………….……………………………….….1  No……………………………………………………2 |
| 21 | Do you have neurovascular ultrasound diagnostic services such as Carotid Doppler Services in the hospital? | Yes ……………….…………………………….…….1  No ……………………………………………………2 |
| 22 | If yes to Q21, can you access this service onsite within 24 hours of stroke presentation to your hospital? | Yes ……………………….………………….……….1  No ……………………………………………………2 |
| 23 | If no to Q21, can you access this service offsite within 24 hours of stroke presentation to your hospital? | Yes ……….……………….………………………….1  No ……………………………………………....……2 |
| 24 | Do you have Electrocardiogram (ECG ) services in the hospital? | Yes …………………….…………………………….1  No ……………………………………………………2 |
| 25 | If yes to Q24, can you access this service onsite within 24 hours of stroke presentation to your hospital? | Yes ……………………….………………………….1  No ……………………………………………………2 |
| 26 | If no to Q24, can you access this service offsite within 24 hours of stroke presentation to your hospital? | Yes ………………………….……………………….1  No ……………………………………………………2 |
| 27 | Do you have Electroencephalogram service in this hospital? | Yes ………………………….……..……….….…….1  No ……………………………………………………2 |
| 28 | If yes to Q27, can you access this service onsite within 24 hours of stroke presentation to your hospital? | Yes ……………….……….………………..…….….1  No …………………………………………..…….…2 |
| 29 | If no to Q27, can you access this service offsite within 24 hours of stroke presentation to your hospital? | Yes ……….……………….………………………….1  No ……………………………………………………2 |
| 30 | Do you have Magnetic Resonance Angiography services in this hospital? | Yes ………………………….…………………….….1  No ……………………………………………………2 |
| 31 | If yes to Q30, can you access this service onsite within 24 hours of stroke presentation to your hospital? | Yes ……………………………………………….….1  No ……………………………………………………2 |
| 32 | If no to Q30, can you access this service offsite within 24 hours of stroke presentation to your hospital? | Yes ………………………….…………………....….1  No ……………………………………………..….…2 |
| 33 | Do you have Computed Tomographic Angiography services in this hospital? | Yes ………………………….……………………….1  No ……………………………………………………2 |
| 34 | If yes to Q33, can you access this service onsite within 24 hours of stroke presentation to your hospital? | Yes ………………………….……………………….1  No ……………………………………………………2 |
| 35 | If no to Q33, can you access this service offsite within 24 hours of stroke presentation to your hospital? | Yes ………………………….………….……………1  No ……………………………………………………2 |
| 36 | Do you have functional acute stroke assessment scales? | Yes ………………………….……………………….1  No ……………………………………………………2 |
| 37 | If yes to Q36, which of the following are used in the hospital? | National Institutes of Health Stroke Scale NIH…..….1  Scandinavian Stroke Scale……………………………2  Canadian Neurological Scale……………………....…3  European Stroke Scale……………….……...………..4  Oxfordshire Community Stroke Project Classification (Bamford)…………………………………...………..5  If others, specify……………………….…….…….…6 |
| 38 | If no to Q36, how do you conduct patient assessment without the use of the recommended tools? | Previous knowledge or experience…….………..……1  Others, specify………………………………………..2 |
| 39 | Does your health service have a dedicated stroke unit (ward)? | Yes …………………………………..………..…….1  No……………………………………….…………..2 |
| 40 | If yes to Q39, is it a | Stand-alone…………………………….……………1  Within another ward……………………………..….2  Combined acute and sub-acute care (e.g. includes rehabilitation) …………………………………….....3  If others, specify …………………………………....4 |
| 41 | If yes to Q39, please describe what this is comprised of | …………………………………………………………  …………………………………………………………  ………………………………………………………… |
| 42 | If yes to Q39, how many beds are in the stroke unit? | ……………………………………………………………..…………………………………………………….. |
| 43 | How adequate are the number of beds? | Very adequate………………………..……………..1  Adequate………………………………………....…2  Inadequate……………………………………..……3  Very inadequate………………………………..…...4 |
| 44 | If no to Q39, dedicated stroke unit, does your hospital have a dedicated, multidisciplinary stroke team? | Yes ………………………………………..…….….1  No ……..…………………………………..…….…2 |
| 45 | If no dedicated stroke unit as asked in Q39, which wards are mostly used to admit stroke patients. | General wards…………………………………….….1  Neurological wards…….……………………….……2  Intensive Care Unit……………………………….….3  Others (specify)…………….………………………..4 |
| 46 | Can you briefly describe what this consists of? | ……………………………………………………………………………………………………………………………………………………………………………… |
| 47 | Which of the following factors account for the lack of or absence of a stroke unit care?  **(Tick where applicable)** | Inadequate stroke clinical staff…………………...…1  Financial constraints ……………………..……...….2  Lack of administrative, policy support ………….….3  unware of the use of stroke unit care ……………….4  If others, specify ……..……………………….…….5 |
| 48 | Will you recommend the provision of a stroke unit in this hospital? | Yes ……….……..………………………..……….....1  No ………..……………………………………..…...2  Not sure………………………..…………………….3 |
| 49 | Is your hospital able to provide thrombolytic therapy using intravenous recombinant tissue plasminogen activator (t-PA or alteplase) for ischemic stroke patients? | Yes ……………………………..………………..….1  No ………………………………..…………………2 |
| 50 | If yes to Q49, is there a standardised protocol or treatment guideline to guide administering of t-PA? | Yes ………………………………………………..….1  No ……………………………………………………2 |
| 51 | If no to Q49, thrombolytic therapy, what is your opinion about the following factors accounting for the non-use of thrombolytic therapy for acute stroke care?  **(Tick where applicable)** | Inadequate stroke clinical staff…………....……….…1  Financial constraints …………………...…………….2  inadequate administrative or policy support ……...….3  unware of the use of thrombolysis …………..……….4  If others, specify ………………….………………….5 |
| 52 | Does your hospital provide aspirin for stroke patients eligible for this type of treatment? | Yes ……………………………………………….….1  No ……………………………………………………2 |
| 53 | If yes to Q52, do you use a standardized protocol when administering aspirin? | Yes …………………………………………..……….1  No ……………………………………………...…….2 |
| 54 | If no to Q52, aspirin therapy, do you agree the following factors account for the non-use of aspirin therapy for acute ischemic stroke care?  **(Tick where applicable)** | Inadequate stroke clinical staff………………………1  Financial constraints ………………………..…….….2  inadequate administrative, policy support ……….…..3  unware of the use of aspirin .………………….….…..4  If others, specify………….………………………..…5 |
| 55 | If no to Q52, would you consider recommending the use of aspirin? | Yes ……………………………………………….….1  No ……………………………………………………2 |
| 56 | Does your hospital carry out surgical treatments for acute stroke patients? | Yes……………………………………………..….…1  No……………………………….…………..……….2 |
| 57 | If yes to Q56, what surgical treatments or procedures are conducted in the hospital? | Revascularization (Carotid Endarterectomy )……..…1  Surgery for Aneurysm…………………………….….2  Arteriovenous malformation treatment……………....3  Decompressive craniotomy………………….…….…4  If others (specify)…………………………...…….….5 |
| **SECTION F: STROKE REHABILITATION SERVICES** | | |
| 58 | Does your hospital have effective rehabilitation services on the same site? | Yes …………………………………………..……….1  No …………………….………..……………….……2 |
| 59 | If no to Q58, on-site rehabilitation service, do you have access to off-site rehabilitation service? | Yes…..…………………………………..……..…….1  No……..………….…..……………….………..……2 |
| 60 | If yes to Q58, please indicate the nature of the rehabilitation service | Public Facility …….………………………………….1  Private Hospital …………………….……………...…2  If others, specify …………………………..…...…….3 |
| 61 | Do you have hospital discharge care plans for stroke patients? | Yes ………………………..…………….………..….1  No ………………………………….…..……………2 |
| 62 | If yes to Q61, what do they include? | Self-management strategies…………………………..1  Out-patient Appointments…….……..……………….2  If others, specify ……………………………………..3 |
| 63 | At discharge, are patients or carers provided with a hospital staff contact number at discharge? | Yes ……………………………....…….…………….1  No ………………………….……………..…………2 |
| 64 | Is there patient information leaflets/literature available/offered on the following topics during admission and at discharge | Stroke condition………………………………………1  Local community care arrangements…………………2  Local voluntary associations………….. …………….3  Community stroke support groups……….………..…4 |
|  | **SECTION G: ORGANISATION OF WORKFORCE** | |
| 65 | How many of the following professionals do you have in your stroke team at your health institution  **(Tick where applicable)** | Clinical psychologist…………………………………1  Nurse …………………………….………..….….…..2  General practitioner………………………...….….…3  Neurologist………………………………...…….…..4  Neurosurgeon………………………………….….…5  Physician specialist………………………….……....6  Medical Officer…………………………………..….7  Stroke care coordinator………………...………........8  Trained stroke nurses…………………………..……9  Emergency department staff……………………...…10  Occupational therapist………………………...….…11  Physiotherapist…………………….………….….….12  Speech pathologist ………………..……..……...…..13  Social worker…………………….………....….…....14  Dietician……………..…………………………...….15  If others, specify ………………..…………………..16  None……………………………………………..….17 |
| 66 | Is there a physician specialist as the principal person for stroke at your hospital? | Yes ……………………………………………..….…1  No ………………………….……………….…..……2 |
| 67 | If yes to Q66, please select one option | Doctor…………………………………………….….1  Nurse…………………………………………….…..2  Therapist………………………………………..……3  If others, specify ………..…………….………..……4 |
|  | **SECTION H: CONTINUING EDUCATIONAND QUALITY IMPROVEMENT** | |
| 68 | Is there a program for the continuing education and professional development of staff on stroke clinical care | Yes …………………………………….………....….1  No …………………………………………..….……2 |
| 69 | Has the stroke team in the hospital been involved in quality improvement activities and on strategies to improve care? | Yes …………………………………………..…..….1  No …………………………….…………….………2 |
|  | **SECTION I: HEALTH SYSTEM POLICY SUPPORT FOR STROKE CLINICAL CARE** | |
| 70 | Are there some specific health policies or interventions (national or hospital specific) meant to improve stroke care? | Yes …………………………………………………..1  No……………………………………………..……..2 |
| 71 | If yes to Q70, which of the following do you have in your hospital? | National health policy for stroke…………………..…1  Hospital interventions and initiatives to support stroke care………………………………………….….….....2  Donor interventions to support stroke care………..….3  If others, specify..…………….…………………..…..4  None……………………………………….…….....…5 |
| 72 | How will you describe the current level of health policy support (oversight) from the national level for acute stroke care? | Limited support…………….…………………………1  Average support……………………………….…..….2  High support.................................................................3  Low support……………………………………….….4  No support……………………………………….…...5 |
| 73 | How will you describe the current level of health policy support (oversight) at the hospital level for acute stroke care? | Limited support………………………………………1  Average support………………………………..…….2  High support................................................................3  Low support………………………………………….4  No support………….………………………………...5 |
| 74 | What do you see as the current limitations of the acute stroke services package? | No stroke unit (ward) ……………...….…….…....…..1  Inadequate stroke clinical staff……………….…....…2  Financial constraints…………………………...…..…3  Health-policy support………...…………………..…..4  Lack of political will………..…………..……………5  If others, specify ….……………..………………..….6 |
| 75 | What plans does the hospital have to promote acute stroke care? | ……………………………………………………………………………………………………………………………………………………………………………… |
|  | **SECTION J: STROKE DATA** | |
| 76 | Do you have a stroke register or database | Yes…………...…………………………………..…..1  No………………………………………………….....2 |
| 77 | If yes to Q76, is it | Electronic stroke data filing system……………….…1  Manual/facility based paper register………….…..….2  None of the above….………………………….……..3  If others, specify...…………….…...…………....……4 |
| 78 | In what ways is the stroke register important? Select where applicable | To identify stroke or other ill-health determinants…...1  To assess the health system performance……….……2  To assess the health status of patients…….………….3  None…………………………………….……………4  Others…………………………………….…………..5 |
| 79 | Are there community or hospital programs/interventions to promote stroke awareness? | Yes …………………………………….……….…….1  No ……………………………………………………2 |
| 80 | Are there community stroke rehabilitative programs? | Yes …………………………………….…………….1  No……………………………………………………2 |
|  | **SECTION K: CONCLUSION** | |
| 81 | Do you have any final comments on any specific section or the interview overall? Any suggestions or ideas?  ……………………………………………………………………………………………………………………………………………………………………………………………………………………………… | |
| 83 | Contact email (optional) |  |
| 84 | Telephone number (optional) |  |
